# Beyond the Clinic Walls: Nurse‐Led Socialised Care and Health Equity for Vulnerable Populations in Australia

**DOI:** 10.1002/nop2.70902

**Published:** 2026-10-14

**Authors:** Kamal Nand Singh, Sonia Martin, Anna McVinish, Apil Gurung

**Affiliations:** ^1^ School of Health University of the Sunshine Coast Moreton Bay Queensland Australia; ^2^ OneBridge Sunshine Coast MC Queensland Australia; ^3^ South Eastern Sydney Local Health District, NSW Health, The Langton Centre NSW Surry Hills New South Wales Australia

**Keywords:** equity, homelessness, social determinants of health, socialised healthcare

## Abstract

**Aim:**

This paper critically examines the Australian College of Nursing's proposed socialised healthcare model and the potential of nurse‐led socialised care as a paradigm shift for addressing the intersection of medical need and social distress among vulnerable populations experiencing homelessness, domestic violence, poverty, and other disadvantage.

**Approach:**

This is a position paper. It offers a theoretical commentary and critical reading that situates the proposed socialised healthcare model within frameworks of social determinants of health, health equity, and socially responsive care delivery.

**Sources and Analysis:**

The paper draws on the illustrative patient narrative presented in the white paper and a critical reading of selected literature on healthcare access and nurse‐led models of care. No systematic search was undertaken. It considered how biomedical approaches may reinforce structural barriers and inequities and examined the potential of nurse‐led socialised models to deliver holistic, person‐centred, and equity‐focused care.

**Discussion:**

We argue that traditional biomedical approaches often fail to address the social and structural factors shaping health outcomes for vulnerable populations. Nurse‐led socialised healthcare models may improve access, continuity, and quality of care through integrated assessment of social determinants, therapeutic relationships, and community‐focused interventions. These models offer a transformative approach to reducing health inequities by recognising health as shaped by social, economic, and environmental factors.

**No Patient or Public Contribution:**

No Patients or Public Contribution.

## 1 Introduction

The intricate relationship between health outcomes and social circumstances has been well‐documented in contemporary health literature. Yet, healthcare systems continue to struggle with operationalising this understanding into effective service delivery models. The Australian College of Nursing's recent white paper, ‘Achieving a Socialised Model of Healthcare in Australia with Nurse Leadership,’ emerges within this contested space, offering both a critique of existing approaches and a compelling vision for transformation (Martin et al. 2024). The analysis that follows is located in the Australian health system. Its funding critique concerns the Medicare Benefits Schedule and bulk billing, and its service exemplars are drawn from Queensland and the Australian Capital Territory, so the specific policy mechanisms discussed are Australian. The underlying argument, that biomedically organised primary care is poorly configured for populations whose health needs arise from social conditions, is not confined to any one jurisdiction.

This commentary positions the white paper's findings within broader theoretical frameworks of social determinants of health, examining how nurse‐led socialised models might fundamentally reimagine healthcare delivery for populations experiencing social distress (Darby et al. 2024). Through critical analysis of the documented patient journey and a critical reading of the evidence, this discussion explores the transformative potential of socialised healthcare approaches whilst acknowledging inherent challenges and limitations.

The narrative that unfolds reveals not merely a critique of current practice, but a fundamental reconceptualisation of healthcare's role in addressing social inequity. As vulnerable populations continue to experience compounding disadvantages through fragmented, medically focused interventions, the urgency for comprehensive reform becomes increasingly apparent (Ojeikere et al. 2021). The urgency for reform is further amplified by rapidly evolving global realities. Climate change, geopolitical instability, and armed conflict are displacing millions of people worldwide, significantly expanding the population of individuals experiencing social distress and healthcare exclusion (Spiegel et al. 2026). Immigrants and asylum seekers represent a growing and critically underserved group within this landscape. Healthcare access for this population varies substantially by legal status. Lawfully present immigrants may access some government‐funded programs, whilst undocumented immigrants are largely restricted to emergency care, community health centres, and limited state‐funded provisions for children and pregnant women (Lebano et al. 2020). Key barriers, including a lack of documentation, fear of deportation, language differences, limited awareness of the health system, and high costs, mirror the structural exclusions that characterise healthcare inequity more broadly (Etemadi et al. 2025). As the global situation continues to increase the number of displaced persons experiencing homelessness, violence, and poverty, any reconceptualisation of healthcare's role in addressing social inequity must explicitly account for this rapidly growing population.

## 2 Theoretical Foundations: Understanding Social Distress Through Healthcare Delivery

### 2.1 The Biomedical Paradigm: Structural Violence and Healthcare Access

The white paper's documentation of Lisa's experience provides a compelling illustration of what Farmer (2004) conceptualised as structural violence, the systematic ways social structures harm individuals through the denial of fundamental human needs. Lisa's journey through the biomedical healthcare system reveals multiple layers of structural barriers that compound her experience of social distress.

The two‐week appointment delay represents more than mere scheduling inefficiency; it embodies a system designed around predictable, non‐urgent medical interventions rather than the complex, immediate needs of vulnerable populations. When healthcare becomes a commodity accessed through market mechanisms, people experiencing homelessness and domestic violence, whose lives are characterised by instability and crisis, find themselves systematically excluded from care (Elliott 2020; Wright 2020).

The ten‐minute consultation timeframe further illustrates the commodification of healthcare relationships. While efficient for addressing discrete medical problems, this temporal constraint fundamentally undermines the relational foundation necessary for understanding and addressing social distress (Green 2024; Tsai et al. 2017). The complexity of Lisa's situation, encompassing housing instability, trauma from domestic violence, medication adherence challenges, and social isolation, cannot be meaningfully addressed within such temporal limitations.

Perhaps most significantly, the financial barriers embedded within the biomedical model create what Sen (1999) described as capability deprivation, a systematic curtailment of the substantive freedoms people require to lead lives they value. Lisa's choice between medication and food represents not an individual failing, but a systematic denial of the capabilities necessary for human flourishing. The healthcare system's failure to recognise and address these structural constraints perpetuates cycles of disadvantage. Structural failures extend beyond citizens experiencing homelessness and domestic violence. Undocumented immigrants and refugees encounter compounded structural violence within biomedical systems, navigating a fragmented landscape of emergency departments, charitable community health centres, and variable government programs in the absence of routine primary care access (Guillot‐Wright et al. 2022). Fear of deportation, language differences, and limited awareness of available services further deter this population from seeking even urgent care. These barriers reflect what Farmer (2004) identified as the systematic denial of fundamental human needs, a denial especially acute for those outside the protections of formal citizenship.

### 2.2 Emergency Care as Social Safety Net: Unintended Consequences

The escalation of Lisa's condition to the emergency department exemplifies the broader phenomenon of emergency care serving as the de facto social safety net for vulnerable populations. This pattern aligns with Lipsky (2010)‘s concept of street‐level bureaucracy, wherein frontline services modify their approaches to accommodate populations insufficiently addressed by formal policy frameworks.

However, emergency departments, designed for acute medical intervention, are structurally unsuited to addressing the social determinants underlying health crises among homeless populations (Walter et al. 2021). The episodic nature of emergency care, focused on stabilisation and discharge, fails to address the underlying social conditions that precipitated the crisis, virtually ensuring repeated presentations and continued deterioration.

## 3 The Socialised Alternative: Theoretical Foundations and Practical Implications

### 3.1 Reimagining Healthcare Relationships: From Transaction to Transformation

The socialised model presented in the white paper embodies what Kleinman (2007) account of moral experience, recognising suffering as fundamentally social and responding through relational engagement rather than technical intervention alone. The immediate accessibility of nurse‐led clinics represents a fundamental shift from market‐based access to healthcare as a human right (Uusitalo 2018). Taylor et al. (2025) reports that extended consultation timeframes enable the attentive, unhurried listening that underpins understanding of how social circumstances manifest as health concerns. For vulnerable populations whose experiences are often dismissed or pathologised, this temporal investment represents both practical necessity and profound respect for their complex realities (Henrickson and Fouché 2017; Scheyvens et al. 2014).

### 3.2 Holistic Assessment: Addressing Root Causes

The comprehensive assessment approach documented in the socialised model reflects Braveman and Gottlieb (2014) a framework for addressing social determinants of health through healthcare delivery. Rather than treating symptoms in isolation, this approach recognises health as emerging from the interaction of biological, psychological, and social factors (Gurewich et al. 2020). The coordination of housing support, mental health services, and community connections exemplifies Antonovsky (1996) a salutogenic orientation to health, in which interventions are designed to enhance the resources and capabilities that assist individuals in managing stress and adversity. For populations affected by homelessness and domestic violence, these capabilities are frequently compromised by structural violence and trauma.

### 3.3 Equity‐Based Care Delivery: Theoretical Foundations

The equity‐based processes documented in existing nurse‐led services reflect Rawls (2017) the difference principle, which holds that social arrangements should be structured to maximally benefit the least advantaged. The five‐step equity process described in Queensland's mobile street health services operationalises this principle through systematic attention to access barriers and culturally responsive care delivery (Martin 2022). This approach acknowledges what Farmer (2004) defined as the fundamental inequality of suffering, the reality that social distress is not randomly distributed but systematically concentrated among populations already experiencing multiple forms of disadvantage. By designing services specifically for vulnerable populations rather than expecting them to navigate mainstream systems, socialised models address structural inequities rather than perpetuating them (Mulvale et al. 2019; Saleheen and Barela 2025).

## 4 Evidence and Outcomes: Empirical Foundations for Transformation

### 4.1 Demonstrating Effectiveness: Existing Initiatives

The white paper's documentation of existing nurse‐led services provides empirical grounding for the theoretical arguments presented above. The school‐based youth health nursing programs demonstrate how early intervention through accessible, relationally based care can interrupt trajectories toward homelessness and social distress (Clare and Rogo 2023; Corney et al. 2021). The mobile street health services OneBridge in Queensland illustrate the rationale for meeting vulnerable populations where they are, both geographically and developmentally (OneBridge 2026). The white paper reports improved health outcomes from these services, attributing this to their attention to the social determinants that traditional healthcare systems ignore or inadequately address. These accounts come from advocacy and service‐provider documentation rather than independent evaluation.

The collaborative General Practitioner–Nurse Practitioner (GP‐NP) partnerships in the Australian Capital Territory (ACT) illustrate how nurse‐led approaches can complement rather than compete with medical care, creating integrated responses to complex social needs. These partnerships demonstrate that socialised approaches need not abandon medical expertise but rather embed it within broader frameworks of social understanding and community engagement (de Leede‐Brunsveld et al. 2023).

The relevance of these nurse‐led models extends equally to immigrant and refugee populations. Mobile street health services and community‐based care, as demonstrated by the OneBridge model in Queensland, are uniquely positioned to reach individuals who cannot or will not access mainstream healthcare facilities (OneBridge 2026). For undocumented immigrants in particular who experience overlapping vulnerabilities of homelessness, violence, and poverty, outreach‐based, culturally responsive nursing care represents one of the few available pathways to meaningful healthcare engagement (Sarpong and Acquah 2026). Nurse‐led services that incorporate multilingual communication, cultural safety frameworks, and non‐judgmental care approaches have demonstrated effectiveness in building trust with marginalised immigrant communities, offering a practical model for extending socialised healthcare to this population (Arias et al. 2026).

### 4.2 Emerging Technologies: Artificial Intelligence as a Complement to Nurse‐Led Care

Alongside structural and relational reform, emerging technologies, particularly artificial intelligence (AI), present significant opportunities to extend the reach of nurse‐led socialised healthcare. AI‐enabled tools can deliver culturally tailored, multilingual health information to vulnerable populations who cannot readily access traditional clinic settings, including undocumented immigrants, homeless individuals, and those in geographically isolated communities (Buchanan et al. 2020). Beyond health education, AI applications in community and public health settings can support triage, early risk identification, and continuity of care through data‐driven pattern recognition. Importantly, AI does not replace the relational foundation of nurse‐led care; rather, it functions as a complementary mechanism that amplifies nursing's capacity to engage with and support vulnerable populations across diverse healthcare settings (Al Khatib and Ndiaye 2025). Thoughtful integration of AI must, however, ensure equity of access, cultural appropriateness, and freedom from algorithmic bias to avoid replicating the very exclusions that socialised models seek to dismantle.

### 4.3 Economic Implications: Cost‐Effectiveness and Resource Allocation

The economic analysis presented in the white paper reveals the false economy of current approaches to vulnerable populations. The Consumer Price Index (CPI) data (Australian Bureau of Statistics 2019) analysed by Richardson (2019) between June 2013 and March 2019, out‐of‐pocket health costs rose 24%, against an 11% rise in the CPI overall, with the increase driven principally by a 33% rise in the cost of medical and hospital services. Increases of this order illustrate how market‐based healthcare delivery excludes those least able to afford care (Martin et al. 2024). The cycle of delayed care leading to emergency presentations represents profound resource misallocation. Emergency departments, among the most expensive healthcare settings, become the primary health providers for vulnerable populations not by design but by systematic exclusion from more appropriate and cost‐effective services (Primm et al. 2019).

The bulk billing model demonstrated in socialised approaches removes financial barriers while offering more comprehensive and ultimately more cost‐effective care. The prevention focuses inherent in addressing social determinants represent what economists describe as upstream investment interventions that prevent more costly downstream effects and consequences (Crowley et al. 2020).

## 5 Critical Analysis: Challenges and Limitations

### 5.1 Structural Constraints: Funding and Sustainability

While the white paper makes compelling arguments for socialised healthcare approaches, critical examination reveals significant structural challenges. The current Medicare Benefits Schedule, organised around discrete medical procedures, provides no mechanism for reimbursing the comprehensive, time‐intensive work that characterises effective care for vulnerable populations. This funding misalignment creates what policy analysts describe as implementation gaps, situations where evidence‐based approaches cannot be sustained within existing institutional frameworks (Gridley et al. 2014). The documented success of existing nurse‐led services often depends on time‐limited grants or organisational goodwill rather than systematic funding commitments.

### 5.2 Workforce Development: Educational and Professional Challenges

Professional preparation for this work requires more than an expanded clinical skill set. It may integrate relevant social‐science perspectives with practical understanding of homelessness and associated chronic illness, mental health conditions, and substance‐use issues; trauma‐informed care; cultural safety; and the health needs of migrants, refugees, and asylum seekers. Preparation may also develop advocacy skills, familiarity with relevant legal and social‐service systems, and the ability to collaborate with interdisciplinary teams and community organisations. Existing work on workforce capacity for homelessness supports the importance of targeted training (Taylor et al. 2025). However, the appropriate content and level of preparation require context‐sensitive evaluation. For roles with sustained responsibility for people experiencing complex social distress, postgraduate or specialist preparation may be warranted.

The socialised model demands nursing competencies that extend well beyond traditional clinical training. Understanding trauma, homelessness, domestic violence, and their intersections requires specialised education that is often absent from standard nursing curricula (Taylor et al. 2025). Furthermore, the emotional labour involved in working with vulnerable populations experiencing social distress requires what Barker (2016) described as emotional competence skills. These skills help manage the psychological demands of bearing witness to suffering and advocating within systems resistant to change.

### 5.3 Scalability and Sustainability: Systemic Transformation Challenges

The documented success of small‐scale nurse‐led initiatives raises questions about scalability. The relational foundation of socialised care depends on continuity and trust‐building that may be difficult to maintain in large‐scale implementations (Boniface et al. 2022). Additionally, the integration challenges between socialised healthcare approaches and existing medical, housing, and social service systems require what organisational theorists describe as system‐level change transformation that extends well beyond healthcare delivery to encompass broader social service coordination (Berta et al. 2014; Harrison and Shortell 2021; Moore et al. 2023).

## 6 Implications for Policy and Practice: Pathways Forward

### 6.1 Systemic Reform: Addressing Structural Barriers

The transformation envisioned in the white paper requires systematic reform of healthcare funding models to recognise and reimburse the comprehensive work necessary for addressing social determinants of health. This might involve developing new billing codes for social determinant assessment and intervention, or alternative funding models that support community‐based, relationship‐centred care.

Policy integration across health, housing, and social services represents another critical reform area. The complex needs of vulnerable populations cannot be addressed through healthcare alone but require coordinated responses that address housing, income support, trauma recovery, and community integration simultaneously. Policy frameworks may intentionally include immigrant populations regardless of documentation. Growing global displacement from climate change, conflict, and instability requires proactive healthcare policies rather than reactive emergency access. Nurse‐led socialised care settings can implement multilingual, culturally safe pathways to address immigrant barriers. Advocating for inclusive, immigration‐sensitive healthcare is both a practical and moral duty aligned with socialised model equity principles.

Policy and practice may also provide structured opportunities for collaboration with civil‐society and community organisations, including faith‐based or religious groups where locally relevant. Such partnerships can help connect clinical care with trusted community networks, practical support, and local knowledge, while retaining clear professional boundaries, shared governance, and attention to equity. Co‐design and ongoing dialogue with community partners can assist services to remain responsive to local priorities (Mulvale et al. 2019).

### 6.2 Educational Transformation: Preparing the Workforce

Nursing education requires fundamental restructuring to prepare practitioners for socialised care delivery. This includes not only knowledge about social determinants of health but practical skills in advocacy, community engagement, trauma‐informed care, and interdisciplinary collaboration. Continuing education for existing practitioners represents an equally important challenge. The transition from biomedical to socialised approaches requires not merely new skills but a fundamental reconceptualisation of professional identity and practice purpose.

This educational transformation may also encompass preparation for the responsible integration of artificial intelligence within nursing practice. As AI tools become increasingly embedded in community health, public health, and outreach settings, nurses require foundational competencies to evaluate, apply, and critically interrogate these technologies. Nursing curricula may incorporate AI literacy not as a peripheral technical skill but as a core component of contemporary socialised care delivery, equipping practitioners to harness AI's potential for reaching vulnerable populations whilst remaining vigilant to its limitations and ethical risks.

### 6.3 Research and Evaluation: Building the Evidence Base

While existing evidence endorses nurse‐led socialised approaches, a comprehensive evaluation of scaled implementations remains imperative. This research must scrutinise not solely health outcomes but also cost‐effectiveness, service user experience, and system‐level impacts. Longitudinal studies investigating the enduring effects of socialised care on vulnerable populations would yield essential data for policy formulation. Such research should utilise methodologies capable of capturing the complex and multidimensional nature of interventions addressing social determinants. Future research may additionally investigate the effectiveness of AI‐nurse collaborative models in public health and community settings, with specific attention to whether AI‐delivered health messaging meaningfully reaches and is understood by vulnerable populations, including immigrants and those with low health literacy.

## 7 Conclusion

The Australian College of Nursing envisions socialised healthcare as a fundamental paradigm shift, challenging current systems that fail society's vulnerable through exclusion and inadequate care. Cases like Lisa's highlight these system failures, demonstrating that biomedical approaches are insufficient in addressing complex social issues. The existing model's emphasis on isolated medical treatments, influenced by market forces and rigid timelines, constructs barriers that exacerbate social distress. This approach is inadequate for populations impacted by social determinants such as homelessness, domestic violence, and poverty. This inadequacy is equally evident for displaced persons and immigrant communities, whose numbers are rapidly increasing in response to global crises of climate change and armed conflict, and who face compounded exclusion from healthcare systems that were never designed with their needs in mind.

Existing nurse‐led initiatives suggest that socialised healthcare is practicable rather than merely aspirational. These models, grounded in principles of equity, relationship‐centred care, and comprehensive services, are reported to improve health outcomes and patient experiences, although independent evaluation of scaled implementations remains limited. On the evidence available, they warrant serious policy consideration and rigorous evaluation. Realising this vision demands transforming funding to equity‐based allocation, curricula to include social determinants, policies to support holistic care, and professional cultures to meet vulnerable populations' needs. Nursing leadership has both opportunity and moral duty, given its size and holistic tradition, to champion equity‐based, socially aware care. The transferability of this argument beyond Australia is uneven. The relational and structural principles, that is, sustained therapeutic contact, outreach to where people are, assessment of social circumstances as clinical data, and nurse‐led coordination across housing, income support, and health services, are portable across health systems. The funding remedies are not: they are framed around the Medicare Benefits Schedule and bulk billing and would require translation into the financing architecture of any other system, whether tax‐funded, social‐insurance, or predominantly private. Systems with weaker universal coverage may find the access barriers described here more acute, while those with established community‐nursing infrastructure may be closer to implementation than Australia currently is. The case set out here is ultimately a normative one as much as an empirical one: systems that exclude those most in need depart from their healing mandate, and reform that recognises the social, biological, and environmental determinants of health is a professional and ethical obligation.

## Author Contributions

**Kamal Nand Singh:** conceptualization, writing – original draft, writing – review and editing, formal analysis, validation, investigation, methodology. **Sonia Martin:** conceptualization, writing – review and editing, methodology. **Anna McVinish:** writing – review and editing, methodology, conceptualization. **Apil Gurung:** conceptualization, methodology, writing – review and editing, investigation.

## Funding

The authors have nothing to report.

## Ethics Statement

The patient narrative referred to as ‘Lisa’ is a published, de‐identified illustrative case reproduced from the Australian College of Nursing white paper (Martin et al. 2024). It does not describe an identifiable patient, and we collected no primary data. Ethical approval was therefore not required.

## Conflicts of Interest

All authors hold voluntary positions within the Street Health Faculty of the Australian College of Nursing (ACN), the professional body that published the white paper examined in this commentary. S.M. is Chair of the Faculty, A.G. is Deputy Chair, and A.M. and K.N.S. provide leadership support. These are unpaid roles held by clinicians working in this field, and no author received payment or other financial benefit for the preparation of this manuscript. The authors disclose these affiliations as a competing interest, given that this commentary evaluates an ACN publication.

## Data Availability

No new data were generated or analysed in the preparation of this commentary.
